# Effects of cognitive training and behavior modification on aggressive behavior and sleep quality in schizophrenia

**DOI:** 10.3389/fpsyt.2024.1363547

**Published:** 2024-05-08

**Authors:** Jing Wang, Gangming Cheng, Hongjie Li, Wei Yang

**Affiliations:** ^1^ Department of Early Intervention, Wuhan Mental Health Center, Wuhan, Hubei, China; ^2^ Department of Mental Rehabilitation, Wuhan Mental Health Center, Wuhan, Hubei, China

**Keywords:** schizophrenia (SCZ), cognitive training, aggressive behavior, sleep quality, sleep

## Abstract

**Background:**

Schizophrenia (SCZ) is linked to a heightened risk of impulsive aggression and disturbances in sleep patterns. Cognitive and social cognitive impairments have been connected to aggression, with social cognitive deficits appearing to play a more immediate role. In this investigation, we conducted a retrospective analysis of the impact of cognitive training and sleep interventions on aggressive behavior and the quality of sleep among individuals with SCZ who were hospitalized.

**Methods:**

This study divided 80 hospitalized patients into two groups according to medical advice, namely the normal group and the study group. The control group received routine drug treatment and education; The research group implemented cognitive training and sleep intervention based on the normal group. Collect basic clinical data, aggressive behavior indicators, and sleep quality indicators.

**Results:**

There is no difference in the basic information statistics between the two groups. Both groups can reduce aggressive behavior and improve sleep quality. In the study group, there was a notable decrease in aggressive behavior compared to the control group. Furthermore, the sleep quality in the study group exhibited significant improvement when compared to the control group.

**Conclusion:**

Cognitive training and sleep intervention have been proven to be effective nonpharmacological treatments, effectively reducing aggressive behavior and improving sleep quality.

## 1 Introduction

Schizophrenia is a mental disorder marked by delusions, speech disorganization, hallucinations, as well as compromised executive functioning (1, 2). With an impact on about 1% of the total global populace, this disorder is ranked as one of the leading 10 factors contributing to global disability. The extent of disturbance SCZ causes in an individual’s daily functioning varies significantly, ranging from high-level functionality in some cases to severe disability in others. In the United States, individuals with this condition face an average potential loss of 28.5 years of life (3–6). Pharmacological and brain imaging studies have demonstrated that disruptions in dopamine neurotransmission can result in psychiatric symptoms, such as delusions and hallucinations, whereas aberrant glutamate signaling may contribute to negative and cognitive symptoms (7–9). Nonetheless, there is an indication that various brain regions and circuits are extensively implicated, leading to a range of effects. Synaptic dysfunction has the potential to induce abnormalities in neuronal connectivity, possibly affecting interneurons, although the precise mechanisms, locations of impact, and timing of these occurrences remain uncertain.

Aggressive conduct observed in individuals diagnosed with SCZ entails the display of destructive behaviors, encompassing verbal and physical assaults toward others, vandalism, as well as self-inflicted harm. These manifestations are frequently prompted by psychotic symptoms and environmental influences (10, 11). Among all types of mental disorders, the occurrence of aggressive behavior is most prevalent in patients with SCZ. Prior research has indicated that around 87.8% of individuals displaying aggression due to mental disorders have received a diagnosis of SCZ (12–15). The occurrence of hostile conduct not only jeopardizes the stability of the patient’s family and societal accord but also significantly impacts the patient’s prognosis and recuperation. Hence, it is crucial to concentrate on hostile conduct in individuals with SCZ and comprehend the factors that contribute to such behavior. This knowledge is essential for implementing efficacious strategies for prevention and early intervention. SCZ is linked to a 4–6 times higher likelihood of engaging in verbal and physical threats, as well as a 4–7 times higher likelihood of committing violent acts when compared to other psychiatric and general population groups (12, 16).

Sleep disorders are prevalent in individuals with SCZ, and there is evidence indicating a connection between sleep disorders and the severity of the condition. Sleep disturbances can manifest as initial symptoms, specifically during the early phase of the disease, before noticeable signs of mental illness emerge. Moreover, they commonly occur during acute psychiatric episodes and have a notable impact on the progression of the disorder (17, 18). The exact nature of the connection between SCZ and sleep remains elusive, and the findings from scientific investigations show discrepancies, potentially resulting from variations in sample characteristics, confounding variables, and disparities in research approaches. Additionally, the medication administered to individuals with the condition might influence the outcomes of sleep-related analyses (19, 20).

In patients diagnosed with SCZ, neurocognitive impairments serve as a distant precursor to violent behavior, whereas social cognitive impairments seem to exert a more immediate influence on everyday acts of aggression (13, 16, 21). There is increasing evidence indicating that deficits in both neurocognitive and social cognitive abilities play a role in the regulation of negative emotions and impulsive aggression among individuals diagnosed with SCZ. Studies have substantiated this claim by examining a group of SCZ patients residing in forensic hospitals, who were randomly allocated to either social cognitive and interactive intervention or a control group receiving general coping skills training. Remarkably, the former group exhibited a significantly lower occurrence of aggressive incidents in comparison to the latter (16, 22, 23). Scientific research has shown that implementing behavioral therapy for individuals with psychiatric conditions leads to a notable reduction in the utilization of sleep medication. Patients diagnosed with SCZ often express a significant lack of understanding concerning proper sleep patterns, highlighting the potential necessity of applying a cognitive behavioral methodology when addressing insomnia within this population (24–26).

This investigation aims to perform a retrospective analysis of the impacts of cognitive training and behavioral intervention regarding aggressive conduct and sleep quality in individuals. Subsequently, we aim to assess the effectiveness of these interventions in alleviating symptoms and anxiety manifestations in patients diagnosed with SCZ.

## 2 Materials and methods

### 2.1 Experimental design

Following the Helsinki Declaration, a retrospective analysis was performed at our hospital within three months, spanning from January 2020 to October 2023. The plan garnered approval from the ethics committee of our hospital.

Divide into a control group and a study group according to intervention measures, with the control group taking conventional drugs to control disease symptoms; based on taking medication, the research group of patients received cognitive training and behavioral intervention. Two groups each have 40 people.

Both patient groups received standard education upon admission, covering topics such as sleep hygiene, daily routines, schizophrenia-related knowledge, medication precautions, signs of disease recurrence, and the importance of medication adherence. Tailored health education approaches were employed based on individual symptoms, including introverted behavior, poor living skills, and lack of social engagement. Positive reinforcement strategies, like work entertainment therapy, were utilized to enhance patients’ self-care, social skills, and interests. Encouraging communication among patients and active participation in rehabilitation training was also emphasized. Patients experiencing uncertainty about the future, lack of confidence, or emotional issues like depression and anxiety were provided with appropriate psychological support. Various techniques were employed to help patients cope with challenges effectively, including correct psychological counseling methods to address emotional disorders and enhance treatment outcomes. Additionally, patients were taught to utilize available resources and develop critical thinking skills for better understanding and evaluation of situations.

### 2.2 Objects

The following criteria were taken into account for study eligibility: (1) individuals aged between 18 and 60 years, (2) diagnosed with SCZ according to DSM-IV criteria or having schizoaffective disorder with an illness duration exceeding 5 years, (3) displaying a rating of five or higher on the aggression items of the Life History of Aggression scale or having committed a confirmed assault within the previous year, (4) obtaining a total score of 15 or higher on the Insomnia Severity Index (ISI), and (5) showing no signs of significant neuropathology or pre-existing intellectual disability based on the patient’s family or guardian’s understanding of the patient’s previous state of life and social behavior.

Patients who were diagnosed with sleep apnea, alcohol use disorder, or substance use disorder were excluded from the study. Throughout the study, all participants were prescribed one or more stable antipsychotic medications.

### 2.3 Intervention methods

#### 2.3.1 Computer-assisted cognitive training

Computer-assisted cognitive training is a personalized software system designed for patients with schizophrenia to improve cognitive function. The system offers a variety of cognitive training exercises targeting different cognitive deficits, starting with basic visual and auditory perception and progressing to neurocognitive and social cognition training. The training program consists of seven parts: introductory practice, perceptual training, attention training, memory training, logical computing training, cognitive flexibility training, and emotional management training. Each part is based on established experimental paradigms in psychology and includes 5 training exercises per session. The first stage of training, spanning 8 sessions over 4 weeks, covers all 7 parts. Before each session, the trainer explains the tasks to the patients to ensure they understand and can effectively complete the exercises. The focus of individualized training is on the 8 training sessions in the second and third stages (weeks 5-8), including personalized training content and adaptive adjustment of training difficulty. The system assesses the level of functional impairment across different cognitive domains based on the patient’s initial 8 training scores. Subsequently, tailored training plans are devised for the final two stages, with additional training provided for cognitive areas that are severely affected. For instance, if a patient’s working memory is more impaired than other cognitive functions, the training content in stages two and three primarily targets working memory. Within each set of 5 training sessions, 3 sessions follow the n-back paradigm, progressing from simpler to more challenging tasks, while the remaining 2 sessions concentrate on working memory training. The content also includes training on other cognitive functions. The training is conducted in a one-on-one tablet format, with trainers addressing any technical issues that may arise during the sessions and promptly documenting any problems encountered. The training approach is gradual and demanding, with the software adjusting the difficulty level in real-time based on the patient’s performance and providing immediate feedback. In the final 8 training sessions, personalized training is further refined based on software recommendations, allowing trainers to adapt the plan according to the patient’s progress and feedback, thereby enhancing the personalized training regimen.

The key features of cognitive training computer-aided system software designed for patients with schizophrenia include standardization, intelligence, personalization, and refinement. Standardization involves the system automatically setting training plans, and expanding the variety of preset plan types. Intelligence ensures that training difficulty adjusts intelligently based on the patient’s cognitive function level. Personalization is achieved through tailored training algorithms that consider user evaluation results, training progress, and age. Refinement allows doctors to manually set and modify preset training plans.

Integrated cognitive training is conducted in strict accordance with the Operation Manual for Integrated Cognitive Rehabilitation Training of Schizophrenia. Quality control of cognitive training interventions is ensured through recording, photography, and other means. Each training session is led by one trainer and one quality control personnel. The quality control personnel primarily assess the quality of cognitive training and provide feedback to the trainers. The research group members hold regular discussions and inspections to enhance the training quality under the supervision of experienced supervisors. The personnel involved in implementing cognitive training and quality control possess the national second-level psychological counselor qualification certificate (including graduate students in psychiatry and mental health, full-time public prevention staff, and full-time rehabilitation staff), while the supervising personnel are chief physicians with extensive intervention experience.

#### 2.3.2 Sleep behavior intervention

Limit the patient’s bed rest time throughout the day, try to approach the actual sleep time as much as possible, and improve sleep efficiency to 80%-90%, thereby improving sleep quality. Based on the principle of conditioned reflex, this treatment is performed. Patients only engage in sleep behavior in bed and bedroom, controlling other stimuli that are not conducive to sleep such as watching TV, mobile phones, and books in bed, establishing positive reflexes that are conducive to sleep, and exerting an inducing effect on sleep. Describe relaxation techniques such as mindfulness breathing and progressive relaxation training, and train the implementation of sleep aid techniques. Further reduces arousal levels, avoids excessive alertness, and alleviates symptoms of psychosomatic disorders. Perform relaxation techniques once a day for 10 minutes each time.

The research group records the patient’s sleep time, bed rest time, wake-up time, frequency, and duration of using relaxation techniques daily. They utilize activity monitors like wrist pedometers to track participants’ activity levels and sleep patterns. These devices offer objective data on bed rest time and actual sleep time, aiding in assessing adherence to bed rest recommendations. A feedback mechanism provides immediate feedback to participants, such as through an app or SMS reminders, to guide them on when to sleep, wake up, and engage in relaxation exercises. This feedback helps participants maintain consistent sleep habits. Interveners offer regular support and guidance to address issues encountered during intervention implementation. By employing these methods, researchers can thoroughly evaluate the compliance of sleep behavior interventions and refine intervention plans to enhance the study’s overall effectiveness.

### 2.4 Monitoring measures

#### 2.4.1 Evaluation of aggressive behaviors

To assess the occurrence and intensity of verbal, physical, self, and object-directed aggression, we utilized the Modified Overt Aggression Scale (MOAS). The MOAS scale comprises four subscales: verbal aggression, assault on property, self-aggression, and physical aggression towards others. It is designed to assess behavior over one week. This scale has been utilized in numerous studies across different diseases, with its effectiveness and reliability consistently validated. Hospital staff members provided eyewitness accounts and coded incidents, compiling data over eight weeks for each participant. Only incidents that were preceded by emotional dysregulation or an inability to manage emotional reactions to provocative stimuli were recorded. Elevated scores on the MOAS reflect heightened levels of aggression. The baseline MOAS data were collected at Baseline, Week 4, and Week 8.

#### 2.4.2 Insomnia severity index

The Insomnia Severity Index (ISI) comprises seven self-rated items that assess the perceived severity of insomnia. These items are rated on a five-point scale, spanning from 0 (not present) to 4 (extremely high), reflecting the perceived level of severity. A higher score implies a more pronounced degree of insomnia severity. The ISI not only evaluates the severity of insomnia but also provides insights into the impact of insomnia on health and daytime functioning. Its user-friendly format makes it easily accessible for patients and has the potential to be a valuable tool for assessing insomnia in clinical settings.

#### 2.4.3 Pittsburgh sleep quality index

The PSQI assesses the quality of sleep and distinguishes between individuals who experience “good” and “poor” sleep. Sleep quality is deemed poorer when global scores exceed 5. For this investigation, the component concerning the utilization of sleep-inducing medication was disregarded during the analysis. The PSQI is a straightforward and reliable tool with strong validity, showing a significant correlation with the outcomes of comprehensive sleep EEG assessments. It is widely utilized in psychiatric clinical settings internationally.

#### 2.4.4 Psychotic symptoms rating scale

The PSYRATS scale is utilized by clinicians to measure the intensity of hallucinations and delusions. It is commonly employed in both research and clinical environments when working with individuals who have psychosis and SCZ. The reliability and validity of PSYRATS are impressive, making it a reliable measurement tool for evaluating both the severity of hallucinations and delusions.

#### 2.4.5 Anxiety sensitivity index

The ASI, which serves as a self-report scale for rating anxiety symptoms, is utilized for quantifying the severity of anxiety symptoms. An increase in scores corresponds to an elevation in anxiety levels. The ASI score is a good predictor of panic to a panicogen.

### 2.5 Statistical analysis

To conduct a comparison of the initial clinical and demographic measures between the control group and study group, a t-test was employed for continuous variables and a chi-square test for categorical variables. Repeated measurements were assessed at various time points to ensure reliability. Covariates included sociodemographic variables with significant differences between groups in the ANCOVA analysis comparing primary and secondary outcome measures at baseline. An ANOVA analyzed interactions between time points (baseline, post-intervention, follow-up) and groups, with post-hoc tests conducted for significant differences. Bonferroni corrections were used to detect group differences at each time point in the ANCOVA analysis. The statistical significance level was established at *P* < 0.05. Data analysis was carried out using IBM SPSS Statistics 26.0 software.

## 3 Results

### 3.1 Participants characteristics

Retrospective analysis was conducted on 60 patients in each of the control group and the study group. Among them, 10 patients in the control group had incomplete information, 5 patients were discharged midway, and 4 patients used other drugs midway, which did not meet the requirements of this experiment. In the final analysis of 40 patients in the control group; 8 people in the research group had incomplete information, 12 people withdrew midway, and 40 people were ultimately analyzed. Initially, there were negligible sociodemographic dissimilarities observed when comparing the study groups with the control groups (**Table 1**).

**Table 1:** Comparison of basic information (n=40).

	control group	study group	t/χ2	*P*
Age (years)	42.70 ± 8.29	45.13 ± 8.16	1.319	0.191
Antipsychotics dosage (mg/d)	10.13 ± 4.48	11.63 ± 4.44	1.504	0.137
Sex			0.457	0.499
male	21	24		
female	19	16		
Duration of illness (years)	7.67 ± 3.60	7.73 ± 3.97	0.074	0.941
Age of onset (years)	35.03 ± 8.39	37.40 ± 8.82	1.228	0.223
Education (years)	11.38 ± 2.37	11.17 ± 2.60	0.359	0.720
Marital status			3.027	0.220
Single	10	12		
Married	15	20		
Divorced/Widowed	15	8		

### 3.2 The impact of implementing measures on attack behavior

**Figure 1:**
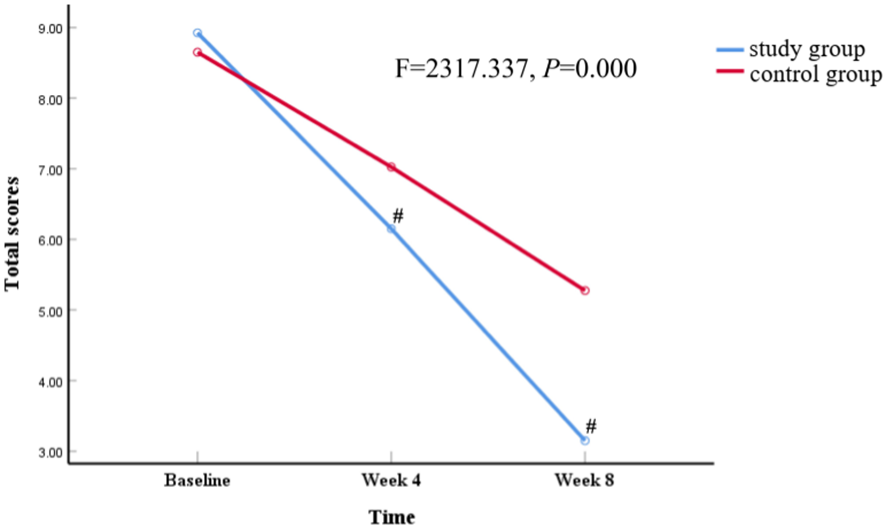
The noteworthy variations in overall scores according to time. ^#^ *P*<0.05, study *VS* control group.

Both groups demonstrated considerable enhancements in verbal assault, property assault, self-inflicted harm, physical assault, overall score, and total number of attacks. The total score exhibited noteworthy time and group interactions, with a significant relationship observed (total scores; F=2317.337, *P* =0.000) (**Figure 1** and **Table 2**). However, no significant difference between Self attack and total number of attacks in group × time interactions, while other indicators showed significant differences (**Table 3**).

**Table 2:** Aggression measures comparison between study and control groups (n=40).

	control group	study group	*t*	*P*
verbal attack				
Baseline	3.22 ± 0.83	3.15 ± 1.46	0.282	0.778
4 weeks	2.40 ± 1.24	2.20 ± 0.82	0.852	0.397
8 weeks	1.08 ± 1.07	0.45 ± 0.55	6.620	0.000
Property attack				
Baseline	3.37 ± 1.43	3.45 ± 1.45	0.223	0.816
4 weeks	2.65 ± 1.21	1.90 ± 1.06	2.952	0.004
8 weeks	1.95 ± 1.01	0.97 ± 0.86	4.640	0.000
Self-attack				
Baseline	1.00 ± 0.93	0.95 ± 0.55	0.291	0.771
4 weeks	0.53 ± 0.64	0.55 ± 0.50	0.194	0.847
8 weeks	0.35 ± 0.53	0.22 ± 0.42	1.161	0.249
Physical Attack				
Baseline	1.87 ± 1.11	1.73 ± 0.93	0.653	0.516
4 weeks	1.22 ± 0.86	1.08 ± 0.57	0.917	0.362
8 weeks	1.02 ± 0.73	0.33 ± 0.47	5.069	0.000
Total score				
Baseline	8.65 ± 1.86	8.65 ± 2.10	0.619	0.538
4 weeks	7.02 ± 1.94	6.15 ± 1.55	2.230	0.029
8 weeks	5.27 ± 1.90	3.15 ± 1.55	5.474	0.000
Total number of attacks				
Baseline	1.35 ± 0.89	1.40 ± 0.93	0.246	0.807
4 weeks	0.83 ± 0.84	0.70 ± 0.69	0.727	0.470
8 weeks	0.53 ± 0.64	0.33 ± 0.53	1.527	0.131

**Table 3:** Attack behavior in time, intervention, and Time-group interaction.

Index	Time		Group		Time* Group	
	F	*P*	F	*P*	F	*P*
verbal attack	411.078	0.000	4.548	0.036	31.401	0.000
Property attack	200.598	0.000	5.787	0.019	9.986	0.000
Self-attack	40.164	0.000	0.177	0.675	1.513	0.227
Physical Attack	79.214	0.000	4.254	0.042	10.880	0.000
Total score	2317.337	0.000	5.836	0.018	160.472	0.000
Total number of attacks	81.851	0.000	0.347	0.558	1.515	0.226

### 3.3 Treatment effects on sleep quality

**Figure 2:**
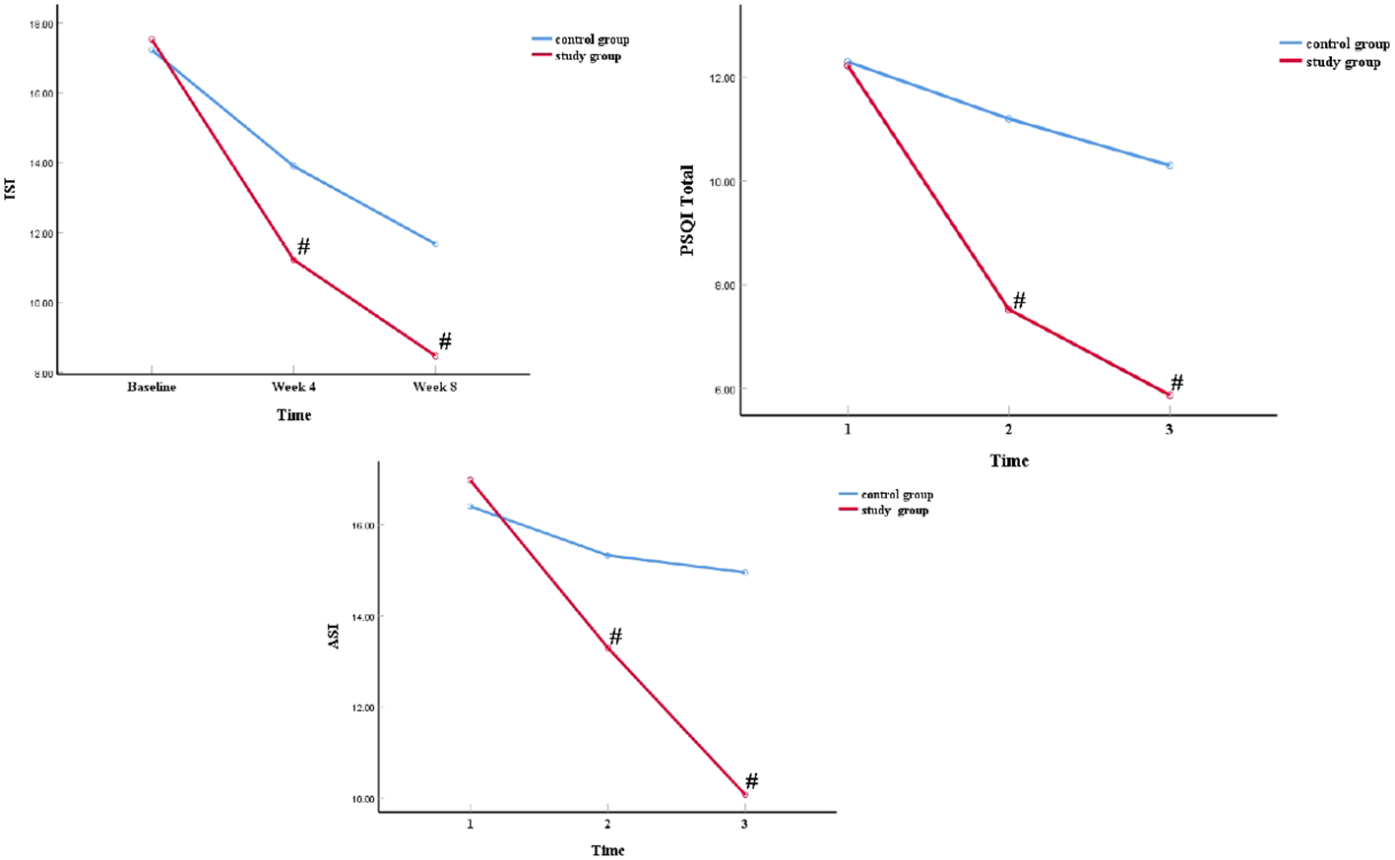
The noteworthy variations in sleep quality according to time. ^#^ *P*<0.05, study *VS* control group.

After treatment, both groups showed significant improvement in the above indicators (ISI, PSQI, PSYRATS, ASI) after management. After cognitive training and sleep intervention measures, there was no difference in the sleep disturbance, PSYRATS total, auditory hallucinations, and delusion indicators compared to the groups. However, no significant difference between sleep disturbance and auditory hallucinations in group × time interactions, while other indicators showed significant differences (**Figure 2** and **Tables 4**, **5**).

**Table 4:** Sleep disturbance comparison between study and control groups (n=40).

Index (time: week)	control group	study group	t	*P*
ISI				
Baseline	17.23 ± 2.54	17.52 ± 2.80	0.502	0.617
4	13.90 ± 2.27	11.22 ± 3.56	4.003	0.000
8	11.67 ± 2.29	8.48 ± 3.23	5.114	0.000
PSQI Total				
Baseline	12.30 ± 1.30	12.22 ± 2.22	0.184	0.855
4	11.20 ± 1.26	7.52 ± 2.49	8.320	0.000
8	10.30 ± 1.76	8.09 ± 2.95	10.145	0.000
Sleep Quality				
Baseline	2.40 ± 0.47	2.43 ± 0.50	0.224	0.823
4	2.30 ± 0.61	1.45 ± 0.50	6.810	0.000
8	2.10 ± 0.74	1.20 ± 0.65	5.767	0.000
Sleep latency				
Baseline	2.35 ± 0.74	2.45 ± 0.90	0.543	0.589
4	2.07 ± 0.66	1.40 ± 0.93	3.756	0.000
8	1.90 ± 0.93	1.03 ± 0.80	4.516	0.000
Sleep duration				
Baseline	1.58 ± 0.50	1.55 ± 0.68	0.188	0.852
4	1.40 ± 0.50	0.58 ± 0.71	6.012	0.000
8	1.25 ± 0.63	0.23 ± 0.42	8.540	0.000
Sleep efficiency				
Baseline	2.40 ± 0.67	2.45 ± 0.63	0.341	0.734
4	2.05 ± 0.75	1.50 ± 0.64	3.529	0.001
8	1.95 ± 0.81	1.15 ± 0.74	4.609	0.000
Sleep disturbance				
Baseline	1.20 ± 0.41	1.15 ± 0.98	0.299	0.765
4	1.13 ± 0.40	1.05 ± 1.01	0.435	0.664
8	1.07 ± 0.42	1.00 ± 0.96	0.453	0.652
Daytime dysfunction				
Baseline	2.38 ± 0.84	2.20 ± 0.79	0.961	0.340
4	2.25 ± 0.81	1.55 ± 0.78	3.934	0.000
8	2.02 ± 0.80	1.27 ± 0.85	4.071	0.000
PSYRATS Total				
Baseline	27.33 ± 1.49	27.37 ± 1.21	0.164	0.655
4	26.27 ± 1.68	26.30 ± 2.17	0.058	0.954
8	25.95 ± 1.57	25.12 ± 2.34	1.850	0.068
Auditory hallucinations				
Baseline	17.45 ± 1.18	17.50 ± 1.06	0.200	0.842
4	17.02 ± 1.31	17.15 ± 1.10	0.462	0.645
8	16.93 ± 1.27	16.83 ± 1.11	0.376	0.708
Delusions				
Baseline	9.87 ± 0.82	9.87 ± 1.78	0.000	1.000
4	9.25 ± 0.93	9.15 ± 2.24	0.261	0.795
8	9.03 ± 0.92	8.37 ± 1.76	2.066	0.042
ASI				
Baseline	16.40 ± 1.24	16.97 ± 2.06	1.515	0.134
4	16.33 ± 1.58	13.30 ± 2.00	5.027	0.000
8	14.95 ± 1.66	10.07 ± 2.19	11.217	0.000

**Table 5:** Sleep disturbance in time, intervention, and Time-group interaction.

Index	Time		Group		Time* Group	
	F	*P*	F	*P*	F	*P*
ISI	1068.898	0.000	9.222	0.003	62.470	0.000
PSQI Total	478.065	0.000	46.882	0.000	151.468	0.000
Sleep Quality	201.085	0.000	22.406	0.000	128.932	0.000
Sleep latency	65.785	0.000	8.259	0.005	20.366	0.000
Sleep duration	187.978	0.000	28.854	0.000	83.897	0.000
Sleep efficiency	156.137	0.000	8.418	0.005	35.296	0.000
Sleep disturbance	4.348	0.016	0.169	0.682	0.058	0.944
Daytime dysfunction	72.892	0.000	9.901	0.002	19.652	0.000
PSYRATS Total	48.728	0.000	0.495	0.484	4.737	0.011
Auditory hallucinations	60.491	0.000	0.010	0.922	1.529	0.223
Delusions	83.679	0.000	0.602	0.440	7.610	0.001
ASI	564.518	0.000	29.302	0.000	274.281	0.000

## 4 Discussion

The emergence, development, and retention of violent behaviors in SCZ are strongly associated with various cognitive impairments. These impairments are closely linked to risk factors such as criminal attitudes, developmental trauma, and prior repeated exposure to violence (27, 28). The presence of global cognitive impairment and absence of insight may serve as substantial predictors of aggression in individuals diagnosed with SCZ. The social functioning of SCZ patients is influenced by both social cognition and neurocognition. In SCZ, social cognition plays a crucial role as an intermediary between neurocognition and incidents of violence. Furthermore, a higher propensity for violence throughout one’s lifetime is significantly linked to alterations in social cognition within the SCZ population. Violent patients with SCZ may exhibit reduced mentalizing abilities (metacognition) in comparison to non-violent patients. In the realm of cognition, various therapeutic approaches have demonstrated their efficacy, such as cognitive remediation and social cognitive training, which have exhibited enduring effects on overall cognition and functioning (29, 30).

Existing evidence indicates that cognitive training can effectively decrease acts of violence and aggression in individuals diagnosed with SCZ (22, 31), The effectiveness of these programs has been scientifically established across different stages of the disease, in a variety of patient profiles, and relation to both aggressive behaviors and verbal and physical assaults. These interventions address not only social cognition, thereby enhancing interpersonal connections and social functioning, but also executive impairments, leading to a decrease in impulsive tendencies. With regards to violence, the aim is to mitigate aggressive behaviors by capitalizing on improvements in executive functions, verbal memory, and social cognition, thereby positively impacting social functioning, including interpersonal relationships, and certain clinical indicators such as impulsivity, excitement, and hostility. In terms of aggressive behavior specifically, our study reaffirmed previous research findings by demonstrating that cognitive training was linked to a significant decrease in impulsive aggression across various metrics. The current investigation yielded noticeable improvements in aggressive behavior within the study group, which aligns with previous research discoveries (16, 32).

Sleep disturbances have been reported in individuals diagnosed with SCZ since the disorder was first described. Traditionally, sleep disturbances have been viewed as a consequence of psychotic symptoms (33). However, sleep dysfunction has been suggested as a contributing factor in the development and continuation of psychotic experiences. The results suggest that addressing sleep disruption can be beneficial in SCZ treatment and that enhancing sleep quality might reduce the intensity of psychosis. Cognitive training stands out as one of the highly successful strategies in managing sleep disturbances (17, 34). A randomized controlled trial was conducted to examine the impact of CBT-I on individuals with insomnia who were diagnosed with psychiatric disorders. Findings indicated a noteworthy reduction in ISI scores within the experimental group in comparison to their initial assessment (35). In their study, Hwang et al. substantiated the efficacy of cognitive behavioral therapy in alleviating insomnia symptoms among patients diagnosed with SCZ. Notably, the intervention demonstrated a sustained impact for 4 weeks following its implementation (36). In the current investigation, the sleep markers exhibited a substantial improvement via week 8 in the experimental cohort, resembling findings from prior research (35, 37). The current investigation exhibited a noteworthy interaction between time and group in terms of ISI, PSQI Total, and ASI. Additionally, subsequent analysis revealed notable disparities among the groups during week 4 or week 8, except for sleep disturbance and auditory hallucinations. These findings are consistent with previous research (36).

To summarize, the current research indicates that the implementation of cognitive training and interventions to enhance sleep quality can offer significant benefits in boosting aggressive behavior and improving sleep quality among individuals diagnosed with SCZ. As scientists delve deeper into the intricate interplay between insomnia and psychiatric symptoms, we anticipate that cognitive training and sleep interventions will eventually prove to be an efficacious approach in mitigating active psychotic symptoms and consequently preventing relapse in SCZ patients. The members of the research group will continue to track participants to evaluate the long-term sustainability of observed aggressive behavior and sleep quality improvement.

## Data availability statement

The original contributions presented in the study are included in the article/supplementary material. Further inquiries can be directed to the corresponding authors.

## Ethics statement

The studies involving humans were approved by the ethics committee of Wuhan Mental Health Center. Written informed consent from the participants was not required in accordance with the national legislation and institutional requirements.

## Author contributions

JW: Investigation, Writing – original draft, Writing – review & editing. GC: Investigation, Writing – original draft, Writing – review & editing. HL: Investigation, Methodology, Project administration, Writing – original draft, Writing – review & editing. WY: Investigation, Writing – original draft, Writing – review & editing.
